# Public sentiment dynamics in policy transitions: a sentiment analysis based on Weibo data

**DOI:** 10.3389/fpubh.2025.1692166

**Published:** 2025-10-31

**Authors:** Xuan Ning, Ruonan Li, Dewei Lan, Chaofan Chen, Yupeng Li

**Affiliations:** ^1^Department of Social Sciences, Beijing Normal-Hong Kong Baptist University, Zhuhai, China; ^2^Faculty of Science and Technology, Beijing Normal-Hong Kong Baptist University, Zhuhai, China; ^3^School of Governance and Policy Science, The Chinese University of Hong Kong, Hong Kong, Hong Kong SAR, China; ^4^Department of Interactive Media, Hong Kong Baptist University, Hong Kong, Hong Kong SAR, China

**Keywords:** COVID-19, health communication, LDA topic model, public health policies, sentiment analysis, Weibo

## Abstract

**Introduction:**

China had been implementing stringent dynamic policies during the COVID-19 pandemic. In late 2022, China made a sudden policy shift from its three-year dynamic zero-COVID to the re-opening policy, which resulted in a divergence of online public opinions and varying sentiments. However, few research has been done to explore the public’s sentiment changes toward this abrupt policy shift.

**Methods:**

To better inform effective health communication regarding governments’ change of policies for future initiatives, this study aims to analyze public’s sentiment changes toward the launching of China’s re-opening policy by using Weibo data. Our study examined 1, 423, 694 Weibo posts during the period from November 11, 2022 to January 11, 2023 to conduct a fine-grained emotion extraction. This study also used the LDA topic model to extract potential topics in Weibo posts to align topics and corresponding emotions for generating in-depth understanding.

**Results:**

Fluctuations of different emotions during these two months were profoundly analyzed and interpreted by taking cultural, social, and policy-related reasons into consideration. Notably, the average proportion of “disgust” (24.0%) exceeded that of “like” (22.8%) after mid-December, while “happiness” exhibited a gradual increase to 12.0%.

**Discussion:**

Results of this study will be essential to informing the government’s effective health communication in the time of public health crisis, facilitating pandemic control and prevention, and enlightening on the maintenance of public’s well-being.

## 1 Introduction

With the global pandemic caused by COVID, nations and states have launched various policies in response to the evolving public health crisis (1). In particular, China has been noted for its adaptive approach to managing the COVID-19 pandemic by dynamically adjusting its policies in line with the changing public health crisis situations (2). China’s dynamic zero-COVID policy, characterized by localized lockdowns and mass testing, initially achieved high containment efficacy (2). However, its abrupt termination in November 2022, marked by *Twenty Measures* policy, created a natural experiment to study public sentiment under deregulation (3, 4). During this period of abrupt changes, the public faced information gaps and adaptation pressure, while government authorities confronted the challenges of maintaining public trust and steering broader public health outcomes. From a crisis communication perspective, such sudden policy shift underscores the challenge of maintaining message consistency and institutional trust when authorities alter their health strategies in a short time span (5).

Over the past 3 years, local governments and communities had actively responded to the state policy and slogan of “avoid gatherings unless necessary” in order to mitigate risks of infection (6). Under this circumstance, communication between people has increasingly transitioned to online platforms. As the primary social media in Mainland, Weibo had been increasingly utilized by both the central and local governments as a channel for disseminating official information and announcements during the COVID-19 pandemic due to its efficient exchange and dissemination of information. Meanwhile, Weibo enables users to post user-generated content and comments, eliciting and facilitating the expression of public opinions (7). When the U-turn policy was launched without dropping any hint to the public in advance, Weibo functioned as a primary outlet for Chinese citizens to express their perceptions and reactions toward this shift. Therefore, during this large-scale public health crisis, Weibo served as a significant bridge in the process of health communication, not only effectively connecting the government, health institutions, and the public (8), but also functioning as a key agenda-setter (9). Through the release of policy information on Weibo, both the authority and media outlets framed the focus of public discourse, while the dissemination of trending topics directly shaped public attention and emotional responses.

Also, it is crucial to acknowledge the significance of health communication, particularly in the context of sudden policy changes during the public health crisis or the period of post-pandemic. From an integrated risk communication perspective, information asymmetry and lack of transparency tend to exacerbate public uncertainty and anxiety (10). Conversely, effective health communication, characterized by timely dissemination of information, can enhance the public’s trust in the governmental and health institutions while improving the public’s health literacy. Especially during policy changes, positive and accurate health communication has the potential to alleviate anxiety and panic.

This study aims to understand the public responses to the abrupt policy changes during the COVID-19 pandemic by analyzing the public’s sentiment changes toward a series of the Chinese government’s re-opening policies by examining over 1 million Weibo posts posted from November 11, 2022 to January 11, 2023. To achieve these objectives, this study uses text emotion extraction method to conduct a fine-grained emotion extraction based on the emotional vocabulary of Information Retrieval Laboratory developed by Dalian University of Technology, which divides human emotions into seven categories and combines Latent Dirichlet Allocation (LDA) topic model to extract potential topics discussed by Weibo users, to get a more complete picture of public opinions. The results of this study will be essential to provide a valuable reference for monitoring public opinions and inform the government’s effective health communication in the time of public health crises, facilitating pandemic control and prevention, and enlightening the maintenance of social harmony and stability. Theoretically, it will extend health communication literature by empirically integrating public emotional responses with established frameworks of crisis communication, agenda-setting theory, and risk communication, thereby offering both practical and conceptual values.

## 2 Literature review

Sentiment analysis (SA), also known as opinion mining, is an ongoing field of natural language processing (NLP) that attempts to determine the computational treatment of sentiment or emotional tone expressed within a piece of text (11). Recently, due to its potential for understanding and tackling societal problems, the study and usage of SA have expanded beyond the discipline of computer science into social sciences. By capturing public attitudes toward specific events, phenomena, and policies, SA assists researchers in gaining a deeper understanding of social trends and shifts in public opinions and group behaviors. In the past decade, the proliferation of big data has resulted in growing applications of SA. The data sources utilized for SA have become increasingly diverse, including news articles, product reviews, and other online resources, which are primarily used to study public opinions and stances for communication, business, and political purposes (12, 13). In these areas, social media data, characterized as user-generated content, namely, original content created by individuals and posted on social media platforms or other channels, are recognized as a valuable resource for SA (11, 14). Such content is typically linked to social networking sites and various social media contexts, and it effectively captures the authentic perspectives and experiences of users, thereby serving as vital materials for emotional analysis (15).

During the COVID-19 pandemic, researchers conducted research on examining people’s attitudes and emotions towards COVID-19 based on data obtained from social media websites, such as Twitter and Weibo (16, 17, 41). Based on the data from social media during the pandemic, many researchers have investigated the psychological trends and attitudes of netizens. For instance, to identify the public sentiments of 12 different countries regarding COVID, Dubey (16) used the NRC Word-Emotion Association Lexicon, which revealed that most people (about 55%) possess negative sentiments toward COVID, and sadness and disgust are the most common sentiments expressed. Shi et al. (17) used the emotional vocabulary of the Information Retrieval Laboratory developed by Dalian University of Technology to examine the developmental course of online public opinion during the first pandemic wave of the COVID-19 pandemic in China by collecting Weibo data from December 1, 2019 to April 30, 2020. Their findings indicated that central cities exhibited a stronger emotional response to the pandemic compared to other urban cities, especially in Wuhan, where the outbreak originated from (17). By mining the data with hashtags, such as #IndiaLockdown, Barkur et al. (18) investigated the opinion of Indians at the time of the lockdown during the pandemic and concluded that the dominant sentiment was positive. In addition, Vijay et al. (19) also analyzed the sentiment of Indians by analyzing tweets containing three keywords (corona, COVID, COVID-19) and concluded that people’s initial sentiments toward lockdown were generally negative, but as time progressed, sentiments gradually shifted to neutral or positive.

Research focus has been shifted to study the public’s sentiment changes as policies launched by governments of different countries began to change together with the gradually stabilized COVID situations, especially countries like China began to lift up restrictions to resume public and cross-border activities. Song et al. (20) conducted an investigation on the public’s emotional responses in Shijiazhuang city from November 13, 2022 to November 23, 2022, with the initial phase of the policy shift. Their findings indicated that there were no significant emotional fluctuations during this period, however, changes in emotions were observed over time. Additionally, Zhang et al. (21) focused on the turning point of both positive and negative emotional evolution and “hot topic searches” events among social media users from November 1, 2022, to March 1, 2023. Their research analyzed the evolving behavioral factors influencing emotional responses, which highlighted behavioral concerns related to disease management, holiday atmosphere, etc.

Even though there is a dearth of research that has been done using SA on social media to study people’s attitudes during the COVID-19 pandemic around the world, including China, little research has been conducted to analyze the sentiment changes during China’s COVID re-opening policy period. Existing research on emotional changes in the context of China’s re-opening policy is limited, typically focusing on the initial stages of policy change and being limited to a specific region (20). Wang et al. (22) have examined public opinions regarding adjustments to public health policy, emphasizing the impact of social derivative events on shifts in public expectations. Their findings indicate that public opinion plays an important role in formulating policies to deal with public health crises (22). While the study conducted by Zhang et al. (21) has identified key turning points in emotional responses and influencing factors, few studies explored refined emotional extraction during the gradual process of China’s re-opening policies. It is seen that existing studies present methodological limitations. Some solely rely on SA, or in an attempt to further identify influencing factors, conducting separate analyses of data on positive and negative emotions (21). This approach fails to adequately capture the complexity of public emotions and their interactive relationship with policy-related issues.

In contrast, the distinct contribution of our study lies in its innovative integration of fine-grained sentiment extraction and topic modeling, with a specific focus on the critical period of abrupt changes in China’s pandemic control. This approach not only captures subtle emotional dynamics such as fear, disgust, and like, but also directly aligns these sentimental shifts with evolving topics discussed on Weibo, thereby revealing how emotions and issues co-evolved during this transition period. As the COVID-19 pandemic was first reported in China, and since China may have relatively the strictest policies and regulations on pandemic control and management, understanding Chinese citizens’ perceptions on the sudden U-turn of the government’s policies is of essence. Therefore, this study aims to study public’s sentiment changes toward a series of re-opening policies launched by the Chinese government since November 11, 2022 to January 11, 2023 to provide insights from the perspective of health communication, contributing to both methodological and theoretical advancements.

## 3 Methodology

### 3.1 Research design

**Figure 1:**
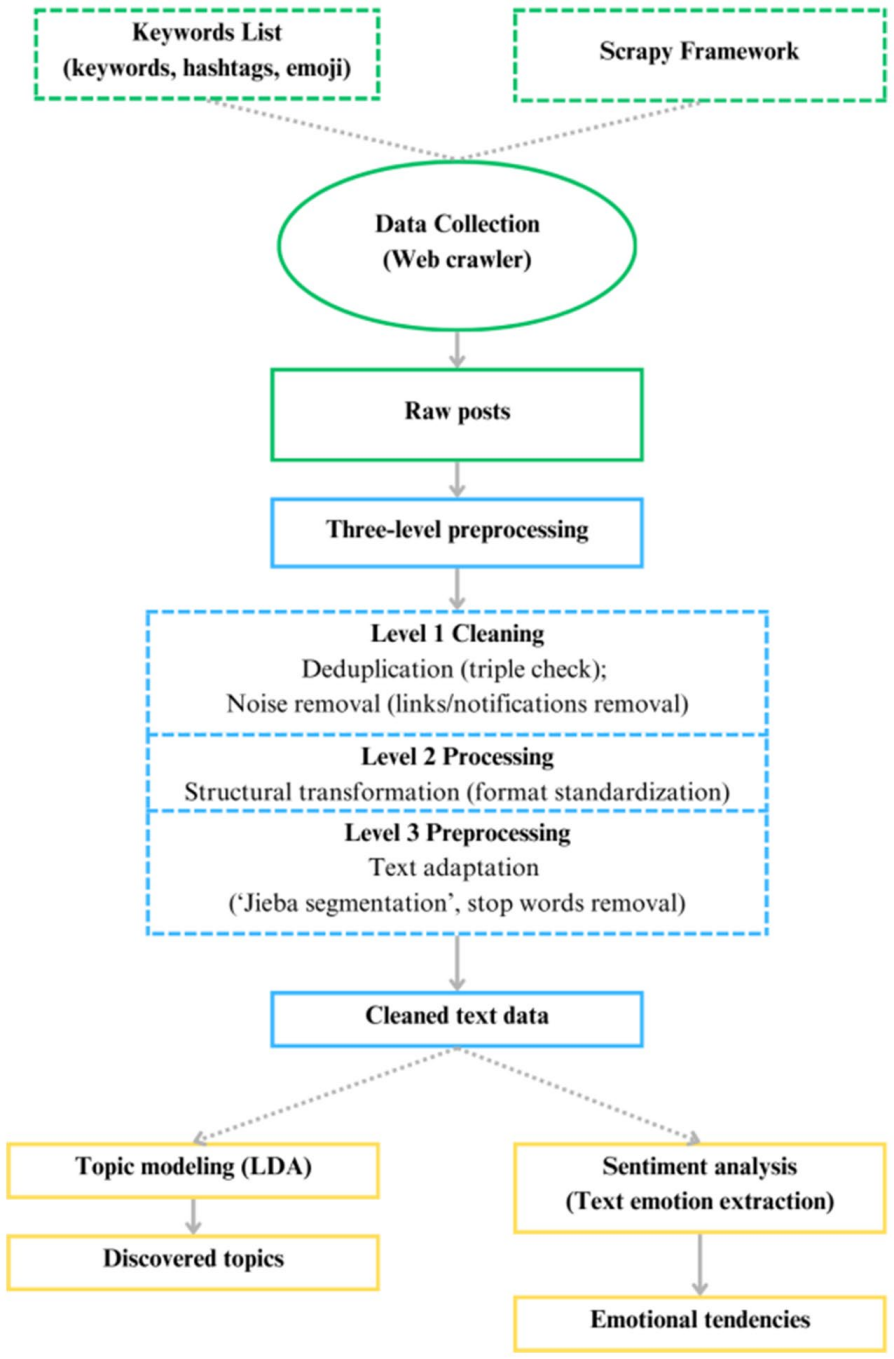
Methodological flowchart.

Figure 1 illustrates the methodological flowchart of this study. The research began with data collection, utilizing a Python web crawler to gather raw posts. These posts then underwent a preprocessing phase, during which we designed and implemented a three-stage data-cleaning pipeline to ensure reproducibility. Following this pipeline, the data were subjected to topic modeling using the LDA model and text emotion extraction. The detailed procedures for data collection and preprocessing are described in the subsequent section.

### 3.2 Data collection and preprocessing

Sina Weibo is the most prominent social media platform in China, with 462 million monthly active users and 130 million daily text exchanges (23). Most posts on Weibo are publicly accessible, and the platform allows for social interactions among users, which provides a valuable source of information for studying attitudes toward launching re-opening policies related to the COVID-19 pandemic. However, it should be acknowledged that Weibo users do not fully represent the broader Chinese population. Active users are disproportionately younger, urban, and digitally engaged, while older adults population is relatively underrepresented (24). This demographic skew implies that the sentiments analyzed in this study may better capture perspectives of urban and digitally literate groups, and findings should therefore be interpreted with this limitation in mind.

To address our data collection task, we utilized a Python web crawler, a currently widely used tool for data collection. It simulates human logins and performs searchers for Weibo posts based on specified keywords, hashtags, and emojis, enabling efficient and consecutive retrieval of nearly all search results for the given keywords (25).

To begin with, we used Python to mine and collect data from Weibo using related hashtags, keywords, emojis (such as sun or sheep, which were used frequently by Chinese netizens to represent testing positive for COVID), and some Chinese characters that are homophonic with nucleic acid and testing positive for COVID in Chinese as filters. Then, a further screening and extraction process was conducted to refine these keywords, resulting in a finalized and relatively complete list of keywords. Among these selected keywords were COVID-19, control, emoji sun or sheep (used frequently by Chinese netizens to represent testing positive for COVID), etc. The complete list of search terms including hashtags, keywords, and emojis is shown in Table 1.

**Table 1:** List of search terms.

Original Chinese hashtags	Corresponding English translation of search hashtags
#优化防控工作的二十条措施	#Twenty measures to optimize prevention and control
#全国多地优化防控措施	#Optimizing COVID-19 prevention and control measures in many parts of the country
#感染新冠怎么办	#What to do if you are infected with COVID-19
#新十条	#New ten measures
#进一步优化防疫新十条	#Further optimization of the new ten measures
#新十条不是全面放开而是主动优化	#New ten measures aren’t completely freeing up the prevention, they are proactive optimization
#做自己健康的第一责任人	#Being the first to take responsibility for one’s own health
#北京又增两例死亡病例	#Two more deaths in Beijing
#目前还没阳性的人的生活状态	#Life status of those who have not tested positive so far
#大概率都会感染越晚变阳症状越轻	#Most people are likely to get infected, and the later they test positive, the milder their symptoms will be

Original Chinese keywords	Corresponding English translation of search keywords
疫情、阳、羊、隔离、感冒、酸痛、核酸、查绿码、行程码、封控、放开、恢复线下、阳性、专家、药、发烧、症状、喉咙、睡觉、抗原、头疼、感染、高峰、老人、小孩、疫苗、加强针、扎堆就诊、神雕侠侣、杨过、杨康、王重阳、复阳、转阴、群体免疫、医疗资源、躺平、小阳人、复苏、精准防疫、科学防疫、中招、感染、轻症、密接、重症、方舱、无症状、硬扛、人没了、退烧药、做好准备、死亡、全部放开、摆烂、基础病、实际感染、奥密克戎、唐飞、囤药、涨价、愿检尽检、撞门、医疗挤兑	COVID-19, sun / positive, sheep, quarantine, cold soreness, nucleic acid, lockdown, check green code, trip code, open, resumption of offline, positive, expert, medicine, fever, symptoms, throat, sleep, antigen, headache, infection, peak, older adults, children, vaccine, booster shot, gather for medical treatment, Divine Condor Hero, Yang Guo, Yang Kang, Wang Chongyang, re-positive, turn-negative, herd immunity, medical resources, lie flat, small-positive people, recovery, precision prevention, scientific prevention, get infected, infected, minor illness, close contact, serious illness, makeshift hospital, asymptomatic, grit it out, the person is gone, antipyretics, be prepared, death, fully open, slack off, underlying disease, actual infection, Omicron, Tang Fei, stockpile medicine, price hike, willingness to self-test, crashing into the door, medical squeeze.

Original search emojis	Corresponding interpretation of emojis
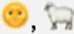	Sun, Sheep
	(In the context of the pandemic, they are commonly used as a homophonic pun to refer to “yang” (positive), or people infected with COVID)

Data collection was conducted using the open-source GitHub project Weibo-search (version 0.3.2). The crawler was implemented with the Scrapy framework, with a minimum interval of 3 s between requests from the same IP address. To mitigate anti-crawling mechanisms, two static proxy IPs were rotated, and multi-process parallelization was applied on a single workstation to enhance efficiency. They retrieved dataset included fields such as posting time, user ID, post content, and engagement metrics. Due to constraints of project duration and computational resources, only “hot Weibo” posts within the specified time frame, from November 11, 2022 to January 11, 2023, were collected as the sample. All data were obtained exclusively from publicly available, in strict compliance with the *Regulations on Network Data Security Management* as well as Weibo’s open API policies, and no user privacy information was accessed.

Then the data underwent a systematic preprocessing procedure (see in Figure 1). In the first stage, duplicate and noisy entries were removed by applying a composite key of user ID, timestamp, and MD5 hash to eliminate repeated posts and by filtering out URLs and system notifications (e.g., “this content has been deleted”). The second stage focused on structural normalization, which involved trimming redundant whitespace and line breaks and removing meaningless formatting symbols such as “#.” Thirdly, the text was adapted for analysis through Jieba segmentation, retaining tokens with two or more Chinese characters, and stopword removal using a widely adopted Chinese stopword list from GitHub. This allowed us to discard punctuation, function words, and low-information internet slang while preserving semantically rich and emotion-bearing terms. Ultimately, we collected a total of 1, 423, 694 Weibo posts from November 11, 2022 to January 11, 2023.

### 3.3 Data analysis

#### 3.3.1 Topic modeling

Topic modeling is considered as a powerful method in text mining, uncovering latent information, and discovering connections among data and text documents (26). Of all the existing topic modeling approaches, including Non-Negative Matrix Factorization (NMF) and Latent Semantic Analysis (LSA), LDA stands out as a prevalent choice (26). LDA posits that each document consists of multiple topics, with each topic represented by a set of keywords (27). It aims to examine the underlying thematic structure within a collection of texts. That is, by analyzing the patterns of word usage in documents, it can facilitate the identification of latent topics and discover the distribution of these topics in the specific corpus. This enables LDA to effectively extract the probability distribution of topics and keywords associated with each topic within the document collection. Hence, to help identify the topics discussed by Weibo users concerning the pandemic during the period surrounding the launching of the re-opening policy, this study employs LDA topic modeling to gain a deeper insight.

The implementation of LDA was carried out using the Python gensim library. Candidate topic numbers ranging from 4 to 19 were evaluated, and the optimal number of topics was determined to be six based on coherence and perplexity scores. Firstly, perplexity can be understood as a measure of predicting or understanding a given set of words or sequence of words. As it is customary in language modeling, perplexity decreases monotonically with the increased likelihood of the test data (28). Simply put, a lower perplexity indicates that the model demonstrates greater confidence in predicting the subsequent word in the sequence, whereas a higher perplexity signifies a less accurate prediction. More formally, its meaning is contained in the following calculation formula:


$$\text{perplexity}\;(D_{\text{test}})=\exp(\frac{\Sigma_{d=1}^{M}\;\log p(\mathrm{wd})}{\Sigma_{d=1}^{M}\;N_{d}})\;$$

where M represents the number of test texts, Nd denotes the number of words in the text, and p(wd) refers to the probability of the text, which is the product of the probabilities of all its words. The probability of each word can be computed using the total probability formula.

In addition to predicting the likelihood of topic distribution by using perplexity, the coherence score is an important metric as well. In the statistical language model, it is used to measure the relevance between texts, which can help to determine the best number of topics. That is, the closer the words are semantically, the higher the consistency score. On a more formal note, the consistency score is calculated by the co-occurrence frequency of the words in the sliding window, and the calculation formula is described as follows (25).


$$C_{U}\;\text{Mass}=\frac{2}{N^{*}(N-1)}\;\sum_{i=2}^{N}\sum_{j=1}^{i-1}\log\;\frac{P\;(w_{i},w_{j})}{P\;(w_{i})}$$

**Figure 2:**
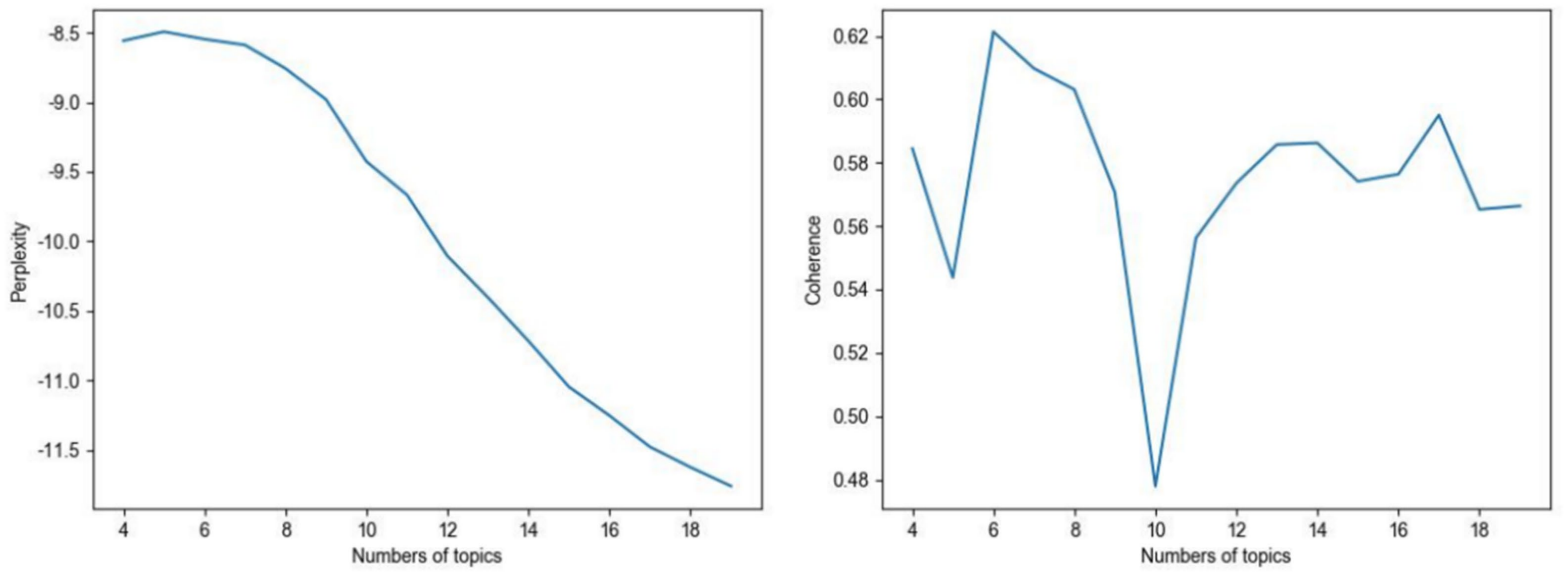
Evaluations of LDA topic model (perplexity and coherence vary with the number of topics).

In practice, there is typically a preference for a model with low perplexity and high coherence. In our study, coherence was regarded as the most decisive metric for determining the optimal number of topics, and we therefore followed the strategy of selecting the model with the highest coherence score (see Figure 2). Notably, the six-topic solution not only achieved this statistical advantages but also generated thematically coherent and interpretable clusters, which further supported its selection as the most suitable model.

In particular, a fixed random seed (random_state = 100) was applied, and documents were processed in batches of 100 (chunksize = 100) with ten training passes (passes = 10). For more detailed analysis, the parameter per_word_topics was enabled. The model was trained using the LdaMulticore function, which allows parallel processing across CPU cores and applies standard symmetric priors. Model evaluation relied primarily on the c_v coherence measure, which captures the semantic similarity of high-probability words within each topic. These parameter settings were chosen to balance interpretability and computational efficiency.

#### 3.3.2 Text emotion extraction

This research adopts a text emotion extraction method, whose main purpose is to determine the emotional nature (such as positive, negative, or neutral) and intensity expressed in the text. It emphasizes extracting fine-grained emotional tendencies from texts. Meanwhile, it follows the general procedure of sentiment analysis, namely, first preprocessing the text, including cleaning the stop words and standardizing the data, Subsequently, emotional vocabulary is used to extract relevant emotional features, followed by the analysis of emotional insight into the text data.

In the concrete analysis of text emotion extraction, this study used the emotional vocabulary of Information Retrieval Laboratory developed by Dalian University of Technology, which divides human emotions into seven categories, namely, like, happiness, anger, sadness, fear, disgust, and surprise. Examples of emotion ontology vocabulary are shown in Table 2. By utilizing the preprocessed textual data as input, matching the words in the lexicon, and tallying the occurrences of emotional keywords, the emotional attributes inherent in Weibo posts can be extracted.

**Table 2:** Examples of emotion ontology vocabulary.

Id	Main category	Category	Example
1	Happiness (乐)	Happy (快乐)	joy (喜悦), delight (欢喜), smiling (笑眯眯), overjoyed (欢天喜地)
2		Peace of mind (安心)	steady (踏实), relieved (宽心), assurance (定心丸), clear conscience (问心无愧)
3	Like (好)	Respect (尊敬)	respectful (恭敬), esteemed (敬爱), full of respect (毕恭毕敬), awed (肃然起敬)
4		Praise (赞扬)	handsome (英俊), excellent (优秀), understanding (通情达理), pragmatic (实事求是)
5		Believe (相信)	trust (信任), confidence (信赖), reliable (可靠), undoubtedly (毋庸置疑)
6		Like (喜欢)	admiration (仰慕), baby (宝贝), love at first sight (一见钟情), infatuated (爱不释手)
7		Wish (祝愿)	desire (渴望), blessing (保佑), long life and prosperity (福寿绵长), endless longevity (万寿无疆)
8	Anger (怒)	Rage (愤怒)	angry (气愤), annoyed (恼火), fuming (大发雷霆), seething with anger (怒气生烟)
9	Sadness (哀)	Sad (悲伤)	sadness (忧伤), sorrow (悲痛), heartbroken (心如刀割), grief-stricken (悲痛欲绝)
10		Disappointed (失望)	regret (懊悔), despair (绝望), discouraged (灰心丧气), disheartened (心灰意冷)
11		Guilty (内疚)	guilt (愧疚), regretful (忏悔), sorry (过意不去), conscience troubled (问心有愧)
12		Miss (思)	miss (思念), yearning (相思), constantly on my mind (朝思暮想), worrying deeply (牵肠挂肚)
13	Fear (惧)	Nervous (慌张)	nervous (慌张), anxious (心慌), at a loss (不知所措), in a flurry (手忙脚乱)
14		Fear (恐惧)	timid (胆怯), fearful (害怕), living in fear (担惊受怕), trembling with fear (胆战心惊)
15		Shy (羞)	blushing (面红耳赤), embarrassed (无地自容), shy (害羞), embarrassed (害臊)
16	Disgust (恶)	Bored (烦闷)	frustrated (懊恼), irritated (烦躁), disturbed (心烦意乱), creating troubles for oneself (自寻烦恼)
17		Disgust (憎恶)	disgusted (反感), shameful (可耻), hatred to the core (恨之入骨), abhor completely (深恶痛绝)
18		Condemnation (责备)	rigid (呆板), vanity (虚荣), chaotic (杂乱无章), ruthless (心狠手辣)
19		Jealousy (嫉妒)	envious (眼红), jealous (嫉妒), jealous person (酸葡萄), jealous of the talented (妒贤嫉能)
20		Suspicion (怀疑)	suspicious (多心), doubtful (生疑), half-believing (将信将疑), paranoid (疑神疑鬼)
21	Surprise (惊)	Surprise (惊奇)	strange (奇怪), miracle (奇迹), astonished (大吃一惊), speechless (瞠目结舌)

To further enhance classification validity, we implemented additional validation steps. While our sentiment analysis relied on the emotional lexicon developed by the Information Retrieval Laboratory at Dalian University of Technology, we examined the keywords list constructed for data collection and manually identified and re-labeled 21 ambiguous terms that could correspond to multiple emotion categories (see Table 3). The sentiment analysis was then run using this refined treatment to reduce classification ambiguity. In addition, we conducted a manual validation of more than 1,000 randomly sampled Weibo posts, assigning sentiment labels independently and comparing then with our lexicon-based classifications using cosine similarity. Although this robustness check provided useful diagnostic insights, the similarity score (=0.1687) was not ideal, which we acknowledge as a limitation of the lexicon-based approach.

**Table 3:** List of 21 ambiguous words.

Original Chinese version of 21 ambiguous words	Corresponding English translation of ambiguous words
杨过, 王重阳,	Yang Guo, Wang Chongyang,
复阳, 中招,	Re-positive, Infected,
杨康, 密接,	Yang Kang (means Turn-negative), Close connection,
人没了, 涨价,	The person is gone, Price hike,
唐飞, 撞门,	Tang Fei, Crashing into the door,
方舱, 硬扛,	Square pods / Makeshift hospital, Grit it out,
阳, 羊,	Positive / Sun, Sheep,
扎堆就诊,	Gather for medical treatment,
摆烂, 躺平, 转阴,	Slack off, Lie flat, Turn-negative,
奥米克戎, 医疗挤兑,	Omicron, Medical squeeze,
囤药	Stockpiling medicine

However, a single text may encompass multiple emotional dimensions. For example, in the sentence, “My nucleic acid turned negative today, very happy and relieved, but the older adults in our family have not recovered, which still makes me panic,” the terms “happy” and “relieved” fall under the category of happiness, resulting in an emotional value of 2 for the happiness dimension. At the same time, the term “panic” functions as a subordinate classification of fear, suggesting an emotional value of 1 for the fear dimension. To ensure that each emotion in the text is adequately represented in the final emotional feature, the extraction uses a one-dimensional vector to indicate emotions. Therefore, the emotional vector resulting from this post-analysis is represented as [‘happiness’: 2, ‘like’: 0, ‘anger’: 0, ‘sadness’: 0, ‘fear’: 1, ‘disgust’: 0, ‘surprise’: 0], which corresponds to the one-dimensional vector is [2,0,0,0,1,0]. Considering that each Weibo post is assigned equal weight, the emotional vector can be normalized using the following equation:


$$V_{\text{normalized}}=\frac{V}{\left\|V\right\|}\;$$

## 4 Results

### 4.1 Emotional changes of Weibo netizens

**Figure 3:**
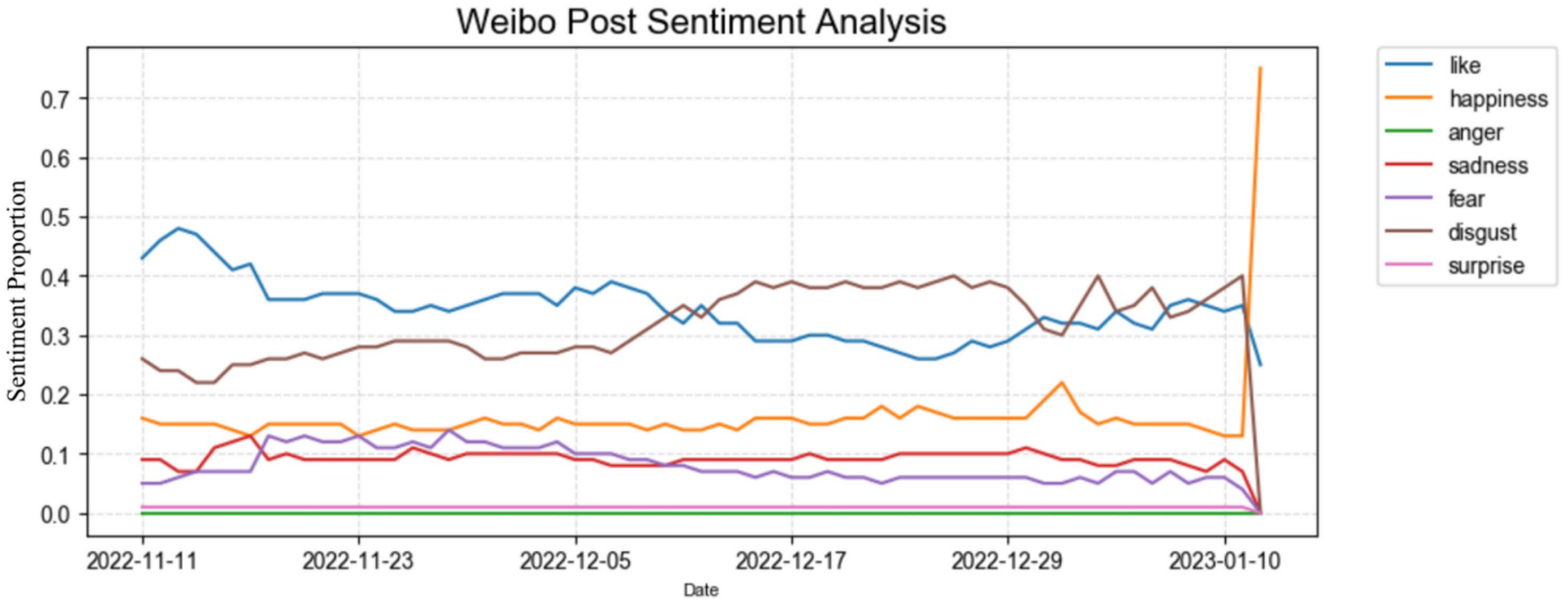
Sentiment analysis on Weibo posts.

To investigate public emotional responses to the abrupt policy shift, we tracked daily fluctuations across seven emotional categories on Weibo from November 11, 2022, when the Chinese government first announced a series of measures to optimize prevention and control strategies (3, 4), to January 11, 2023, 2 months after the series of re-opening policies launched by the Chinese government. Figure 3 shows the emotional changes of Chinese netizens during this period.

As shown in Figure 3, the distribution of public emotions embodies notable distinctions. Particularly, the observation of emotional changes over time revealed that mixed and opposing emotions of ‘like’ and ‘disgust’ predominated other emotions, with ‘like’ occupying the highest proportion while emotions of ‘surprise’ and ‘anger’ were relatively rare (see Figure 3). This phenomenon may well be caused by certain latent reasons, wherein related hashtags and keywords may be classified as belonging to other alternative emotional categories, despite expressing similar sentimental tendencies. In addition, the changes in the seven emotions exhibited distinct patterns, but all of the emotional categories remained relatively stable throughout the observation period. Some substantial fluctuations and peak periods were observed at specific time points. To further ascertain whether these observed fluctuations were more than random noise, we conducted statistical significance tests across key policy stages. As the data did not conform to a normal distribution (Shapiro–Wilk test, *p < 0.001*), we employed the non-parametric Mann–Whitney U test. The results revealed *p*-values far below the 0.05 threshold for comparisons between key stages (December 7–12, 2022, the “*Ten New Measures*” window; and December 13–31, 2022, the period after the travel code was removed), indicating that the changes in emotions were statistically significant. This confirms that the fluctuations observed in Figure 3 reflect genuine shifts in public sentiment rather than random variations.

Specifically, the emotion of “disgust” surpassed the emotion of “like” around December 15, 2022, when “disgust” accounted for 39.0% while “like” dropped to 29.0%. Although the changes of these two emotions were intertwined during the subsequent half-month, the predominance of “disgust” over “like” persisted until December 30, 2022, which implies that this period warrants significant attention due to its notable emotional dynamics (see Figure 3). Besides, the emotion of “happiness” has exhibited a steady upward trend over time. Beginning at 16.0% on November 11, 2022, it gradually rose to 75.0% by January 11, 2023, coinciding with a sharp downward trend in the emotion of “disgust,” as also marked in Figure 3. The specific changes in each emotion, as well as the distinct emotional peak stages, will be explained in detail below.

Initially, following the State Council issued the *Twenty Measures* on November 11, 2022, which simplified risk area classification and quarantine protocols (3, 4), there was a discernible shift in public sentiment on Weibo. On that day, “like” accounted for 43%, while “fear” remained at 5% and “disgust” stood at 26%. The detailed content of *Twenty Measures* involve revising the risk area categorization from “high, medium, and low” to a binary classification of “high and low,” also reducing the number of management personnel. With the implementation of this policy, the “like” mood gradually declined, whereas the “fear” mood rose accordingly (see Figure 3). This dynamic may be attributed to the sudden relaxation of strict public health controls, including the adjustment of several measures from “centralized quarantine” to “home quarantine” meanwhile shortening the quarantine period from 7 days to 5 days. Within two or 3 days following the policy’s implementation, the ease of restrictions garnered positive responses from certain netizens, subsequently leading to a transient increase in the “like” emotion (see Figure 3).

However, in the following days, negative responses potentially characterized this stage, and both ‘fear” and “disgust” had increased. This might stem from the fact that after the policy shift, there was a noticeable escalation in the public’s concern widely, inciting discussions, debates, and disputes on Weibo. Among them, some netizens expressed concerns about relaxing pandemic management, particularly regarding the health problems of vulnerable groups such as the older adults, children, and pregnant women. Additionally, some indicated they were apprehensive about entering public spaces in the near term.

Such negative emotions (including “fear” and “disgust”) continued to fluctuate and rise over time, and then they exhibited a change at the end of November. On November 30, 2022, “like” reached 36%, “fear” rose to 12%, and “disgust” remained stable at 26%, illustrating that while concerns persisted, expectation also began to grow. In late November, when districts in Guangzhou began to issue notifications to remove temporary control measures, there was a noticeable increase in positive sentiments and expressions of satisfaction among the public on Weibo. Over the course of approximately 1 month post the launch of the *Twenty Measures*, there had been a discernible shift in public sentiment. It can be observed in Figure 3 that the emotions of “disgust” and “fear” exhibited a period of fluctuation and rise, ultimately stabilizing at the end of November, concurrently, there was a rise in the emotion of “like.” This trend suggested that concerns regarding the gradual relaxation of restrictions had decreased, giving way to expectations among netizens.

During the first half of December 2022, people’s emotional fluctuations demonstrated increasing volatility. Notably, since December 7, 2022, an upward trend in “disgust” had been observed, surpassing the prevalence of “like” sentiment around December 15, 2022 (see Figure 3). This occurrence may be due to the State Council’s release of the *Ten New Measures* on December 7, 2022, which indicated a further shift in the country’s approach to managing COVID-19, including the further relaxation of lockdowns and public health adaptation. Among these measures, the optimized quarantine mode of “quick quarantine, quick releases” and the promotion of vaccination were included. Moreover, the travel code had gone offline on December 13, 2022, which marked the latest move to dismantle China’s COVID-19 prevention and control infrastructure (29). It was signaled by the transformation in public perceptions, characterized by heightened apprehension toward the soon-fully opened circumstances given the still-existing virus. This time coincided with an escalating infection rate. Since December, both the nucleic acid positivity rate and the number of fever outpatients in the country had increased, surpassing 2 million and 1 million cases by mid-December respectively, and the number had continued to rise thereafter (30), further exacerbating people’s negative emotions.

Subsequently, the “disgust” sentiment persisted at a high level until January (see Figure 3). During this period, the positive rate of antigen detection surged to 1.89 million cases on December 19, 2022, the number of nucleic acid-positive individuals reached a peak (6.94 million) on December 22, while the number of fever outpatients peaked at 2.867 million on December 23 (30), all of which prompted netizens to share personal experiences on Weibo and seek information on how to recover quickly and alleviate symptoms. This rise in the number of cases had led to an increased demand for medications and medical supplies, such as Lianhua Qingwen and masks. Consequently, some medicines were out of stock, and prices had escalated. While a minority of people were hoarding medicines, the majority of netizens reported difficulties in obtaining necessary medications, which reflected people’s anxiety and concern regarding health management in the period of rapid policy change. At the same time, the national postgraduate examination triggered heightened discussions on Weibo. With the influence of high infection rates, thus, candidates encountered the dual challenges of peak infection rates and prolonged anxiety concerning their ability to undertake examinations properly during the arduous preparation phases. These stressors likely acted as potent catalysts fueling the negative emotions among the populace. Moreover, a comprehensive management program for novel coronavirus infections announced by the National Health Commission of the People’s Republic of China on November 26, sparked discussions among the public. The plan indicated that from January 8, 2023, the policy of “Class B infectious diseases” (乙类乙管) policy will be enforced (3, 4), encompassing the cessation of quarantine protocols for people infected, the discontinuation of close contact tracing, and the exemption of inbound people and goods from infectious disease management measures, among other further measures. The announcement of this program sustained concerns and negative emotions of worry.

In the early January, “like” and “disgust” alternated in dominance, with their curves repeatedly crossing each other (see Figure 3). This fluctuation illustrates the ambivalence of public emotions, that is, people were both looking forward to the easing of the crisis and reunion during the upcoming new year and Spring Festival, while at the same time fearing the uncertainties brought by high infection rates and strained medical resources. Furthermore, with the approaching of Chinese New Year within 10 days--events carrying deep cultural and symbolic meaning, “happiness” emotion surged dramatically, culminating in the highest peak across the entire observation period. After 3 years of pandemic restrictions, this Chinese New Year represented a valuable opportunity for people to reunite and embrace new beginnings. This phase, therefore, can be characterized as one dominated by positive sentiments.

Interestingly, the sentiments of anger and surprise remained consistently at a very low and stable level throughout this transitional period. We propose that the stability of these two negative-trend sentiments is influenced by the censorship system of China, which aims to control posts that could have educed unintended negative social consequences. This possible factor may play a role in stabilizing significant fluctuations in “anger” and “surprise” sentiments.

### 4.2 Combined with topic modeling results

To enhance the efficient processing and systematic organization of the extensive textual data obtained through our web crawling, the LDA topic model was employed to categorize Weibo posts from November 11, 2022, to January 11, 2023. Figure 2 shows the curves of posts’ perplexity as a function of the number of topics, alongside the coherence score, for varying numbers of topics. It is observed that the perplexity reaches a peak at approximately 6 topics before gradually declining as the number of topics increases. Additionally, the coherence score exhibits considerable fluctuations under different number of topics, peaking at around 6, and a minimum of 10 topics (see Figure 2).

**Figure 4:**
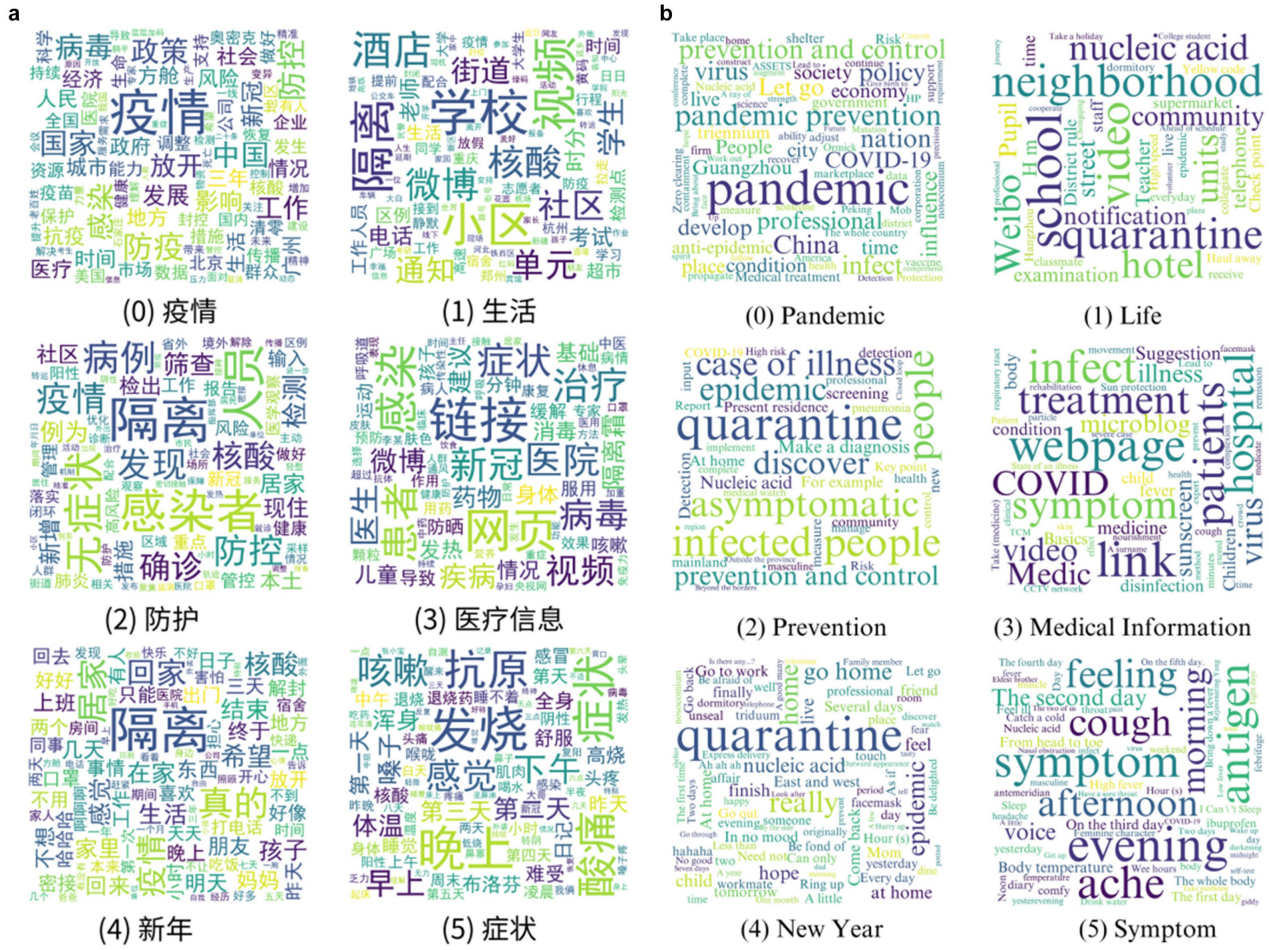
**(a)** Original Chinese word cloud of Weibo posts in different categories. **(b)** corresponding English translation of word cloud of Weibo posts in different categories (it visualizes high-frequency keywords in world clouds, where larger font size indicates higher frequency within the dataset).

An LDA model with 6 topics was selected, and keywords were retained for further analysis. The results displayed in Table 4 was based on the model’s superior coherence score and perplexity (see Figure 2). The topic names were determined by the meanings of keywords, indicating potential topics for discourses within the Weibo discussion. Besides, Figures 4a,b depict the word cloud visualization of the keywords (original Chinese word cloud and corresponding English translation), thereby enhancing the comprehension of the thematic and authentic elements within the text.

**Table 4:** Topics-keywords distribution of posts over Time.

No.	Topic	Keywords
0	Pandemic	pandemic(疫情), pandemic prevention (防疫), prevention and control (防控), infect (感染), policy (政策), virus (病毒), nation (国家), China (中国)
1	Life	school (学校), neighborhood (小区), video (视频), quarantine (隔离), hotel (酒店), nucleic acid (核酸), Weibo (微博), units (单元)
2	Prevention	quarantine (隔离), infected people (感染者), people (人员), asymptomatic (无症状), case (of illness) (病例), epidemic (疫情), discover (发现), prevention and control (防控)
3	Medical information	link(链接), webpage(网页), infect (感染), patients(患者), symptom(症状), (治疗), hospital (医院), COVID (新冠)
4	New Year	quarantine (隔离), really (真的), home (居家), epidemic (疫情), go home (回家), nucleic acid (核酸), hope (希望), at home (在家)
5	Symptom	fever (发烧), evening (晚上), symptom (症状), ache (酸痛), antigen (抗原), cough (咳嗽), feeling (感觉), afternoon (下午)

In the topic modeling analysis applied to Weibo posts, it is observed that the distribution of keywords across categories tends to be relatively consistent, although the relative proportion of each keyword may vary within each category (see Table 4).

Topic modeling of Category 0 identifies dominant lexical clusters, including “prevention and control,” “nation,” and “policy,” which collectively underscore public engagement with national pandemic governance frameworks. Category 1 is comprehensively refined into “life,” emphasizing keywords related to pandemic-induced disruptions to quotidian routines, such as neighborhood containment protocols, and workplace adaptations. For instance, recurrent mentions of “nucleic acid detection” within this category illustrate its normalization as a daily practice. Category 2 centers on prophylactic measures, with high-frequency terms like “case of illness” indicating discourse priorities around risk mitigation. In Category 3, keywords such as “link” and “webpage” signals public reliance on digital platforms for medical information dissemination. The heightened frequency of use of the word “quarantine” in Category 4, combined with the temporal alignment of the research period with the advent of New Year’s Day, suggests people’s concerns about quarantine policies after returning to their hometown. Moreover, keywords such as “hope,” “go home,” and “at home” highlight people’s promising vision; hence this category has been distilled into “New Year.” Finally, Category 5 delineates symptom, centric discourse, dominated by clinically salient descriptors including “fever,” “ache” and “cough,” reflecting lay epidemiology during infection surges.

It can be seen that the implementation of *Twenty Measures* and the subsequent series of policies have significantly influenced routine prevention and control measures in people’s daily lives. Furthermore, the development and prevention measures of the pandemic, symptoms, and accessibility of medical information are the aspects that netizens highly focused on. However, regrettably, the keywords remaining from our data do not conclusively give a clear picture of the mood of the population. But it is enough to understand that the predominant concern during this period is still on the prevention and management of the pandemic itself.

### 4.3 Distribution of emotions category

**Figure 5:**
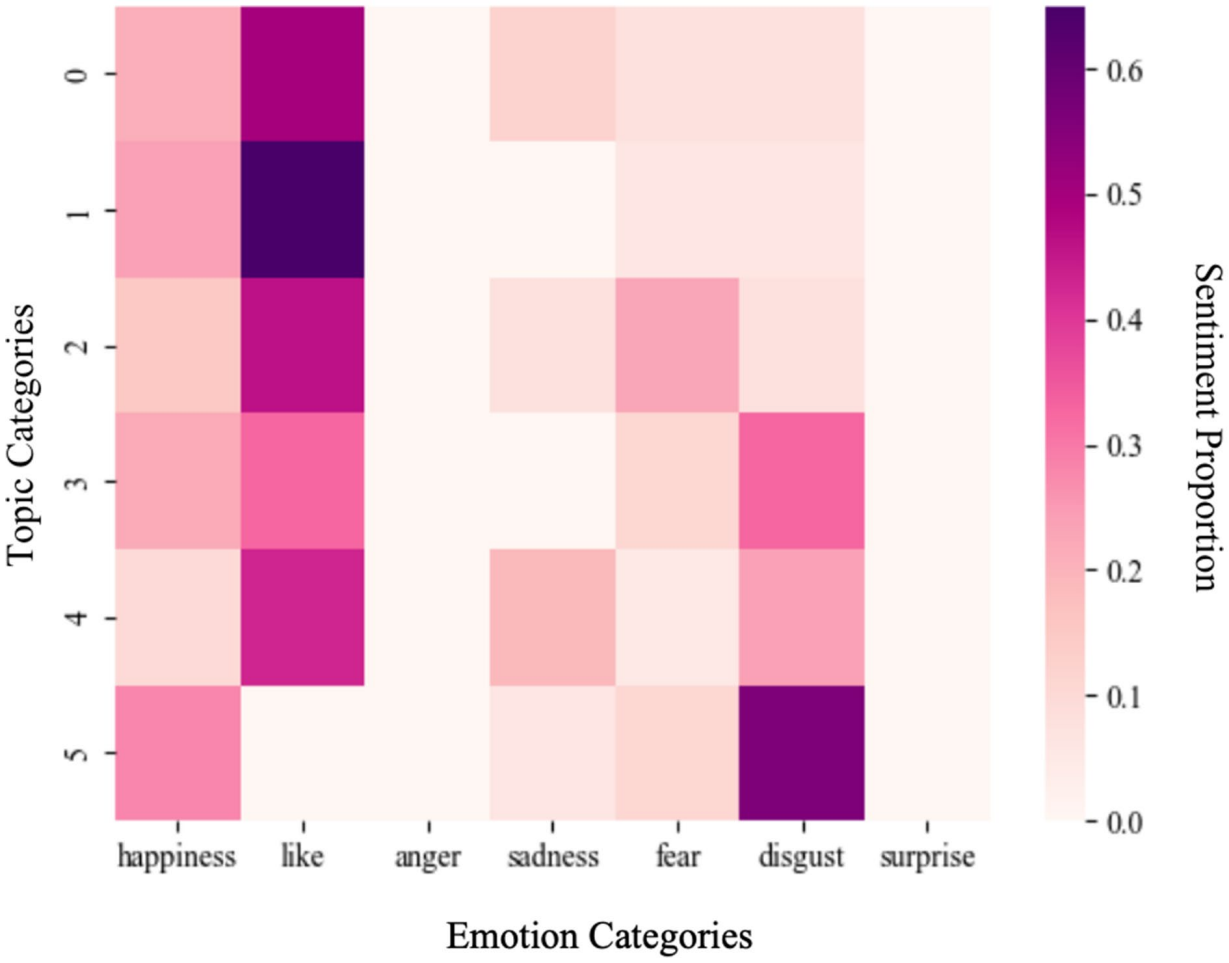
Distribution of emotions category (each bar shows the proportion of seven emotions within a specific topic category, allowing direct comparison of emotional distributions across topics).

To further understand the relative intensity and distribution of emotions within distinct topic categories and reveal people’s emotional trends toward specific Weibo topics related to the pandemic, Figure 5 presents the emotional distribution of different topic categories for posts.

To begin with, the distribution of emotions across various topic categories reveals that the emotion of “anger” and “surprise” consistently appears at a low level, regardless of the category. Meanwhile, the sentiment of “fear” and “sadness” also remain relatively infrequent. By contrast, categories 0, 1, 2, and 4 exhibit a higher prevalence of “like” emotion, particularly in Category 0 (pandemic) and Category 1 (life). In these categories, the topics and keywords distribution (see Table 4) reveals a heightened frequency of “pandemic” and “school,” suggesting a generally positive public attitude towards national-level pandemic polices and daily-life adjustments. In contrast, Category 3 (medical information) and 5 (symptoms) show a higher proportion of “disgust,” which may reflect the public’s frustration with unreliable medical information, treatment difficulties, and negative experiences during infection surges such as fever and cough. Interestingly, Category 4, focused on New Year-related discussions, presents a mixture of both positive and negative emotions. This reflects the dual sentiment of hop and concern: on one hand, anticipation for family reunions during the first post-lockdown Chinese New Year; on the other, anxiety over continued policy adjustments and potential infection risks.

Further analysis of associated keywords confirms a surge in Weibo posts related to “how to prevent” and “what to do” between November 2022 to January 2023, particularly following the release of the *Twenty Measures* on November 11, 2022. This period witnessed heightened public concern, with individuals seeking guidance on protective measures and symptom management. At the same time, growing discussions about the anticipated “post-pandemic lifestyle” also emerged, illustrating the coexistence of both optimism and unease in public sentiment regarding the epidemic and its aftermath.

## 5 Discussion

This study advances the literature on policy communication by exploring public opinions towards the Chinese government’s adjustments of departing from its three-year dynamic zero-COVID policy and to an abrupt re-opening policy shift. To fill the previous research gaps about Chinese citizens’ attitudes during the COVID re-opening policy period, this study examines the fine-grained emotion changes and potential topics discussed based on Weibo posts. Using text emotion extraction combined with LDA topic model, our research analyzed public’s sentiment changes and underlying topics toward the Chinese government’s re-opening policy adjustment from November 11, 2022 to January 11, 2023. It should be noted that the results we obtained were derived from Weibo users, they were likely to over-represent urban, younger, digitally engaged, and digitally less engaged populations. Future studies should triangulate across multiple platforms or offline surveys to assess broader groups of people in order to increase the representability of our results to understand the public sentiments.

Our findings indicate that public opinions on policy changes have experienced emotional fluctuations, reflecting the evolution of public perceptions. The emotional responses observed in the initial stages of policy change in this research align with previous research, substantiating that while positive emotions tend to remain stable in the early stage of policy change (21). However, this stabilization proved short-lived, as operational uncertainties—including medical resource accessibility and infection risks—precipitated a rapid inversion of sentiment. Negative emotional indices, particularly “disgust” and “fear,” demonstrated progressive intensification from late November 2022 onward, diverging from documented patterns of gradual emotional normalization in incremental policy transitions (21). Importantly, our results show that “disgust” dominate other emotions rather than “fear,” because it was more closely tied to tangible daily frustrations, such as shortage of medicine, and policy inconsistencies, which elicited stronger aversive reactions than abstract fear of infection itself. From a communication perspective, this pattern resonates with situational crisis communication theory, which emphasizes that public emotions depend on perceived responsibility attribution (5). While fear reflects a natural response to health risks, disgust emerges when citizens hold institutions accountable for policy failures and unmet expectations. In this sense, disgust was not only an individual emotional reaction but also a communicative signal of broke trust in crisis management, explaining why it became more dominant than fear during this period.

This sentiment disjunction phenomenon emerged most starkly during rapid deregulation phases, exemplified by the sudden termination of travel code systems—a measure that disrupted populations habituated to prolonged containment protocols. After that, the implementation of the *Ten New Measures* on December 7, 2022, further amplified this dissonance, as medication shortages and supply-chain disruptions coincided with heightened public dissatisfaction. This finding echoes previous research positing that abrupt policy reversals amplify implementation-related anxieties (21). Furthermore, the subsequent resurgence of positive affect in January 2023 correlated temporally with declining infection rate and Chinese New Year tradition, underscoring cultural and temporal moderators’ capacity to mitigate policy-driven emotional volatility. Comparative perspectives also highlight that China’s sentiment trajectory during the re-opening phase shares both commonalities and differences with other national contexts. In the United States, studies show that while fear dominated in early lockdown, public emotions shifted toward more positive and supportive tones once re-opening began. This could be partly explained by strong economic pressures to resume employment, cultural expectations of returning to normal life, and the framing of re-opening as a pathway to recovery (31, 32). By contrast, in India, weaker health infrastructure and socioeconomic vulnerabilities amplified fear and anger during the Unlock phases (33). These cross-country comparisons suggest that sudden policy shifts often elicit different sentiments, which cannot be understood in isolation but must be contextualized within each country’s real-world changes, such as infection surges, cultural, political, and institutional settings, as well as the communication channels.

Our research discovers a range of topics discussed on Weibo, as identified through LDA topic modeling. These findings indicate that individuals focus not only on the pandemic itself and the measures for controlling and preventing COVID-19, but also on the repercussions in the wake of the pandemic and the influence on their daily lives. Culture tended to interplay with the pandemic to affect the public’s sentiments. There were heated discussions on returning to the hometown for New Year celebration, which spurred strong emotional responses among individuals, as reuniting with family members on Chinese New Year was of critical cultural and symbolic meaning to the Chinese. Among these topics, it is also noteworthy that the discussions surrounding symptoms and medical information were heated as well, as both represent critical content of health communication (34). The accurate dissemination and exchange of such information are essential for guiding strategic treatment decisions. Combined with the results of emotion extraction and distribution of emotions category in distinct topics, using the topic of medical information as an example, it is observed that during the period of medical supply shortage accompanied by the increase of price, people show negative responses, meanwhile, the distribution of emotional categories underscores the importance of disseminating medical information online implied by the keywords “link” and “webpage” under the medical information topic category. Therefore, it can be postulated that when discussing such topics, institutions related to healthcare need to make efforts on social media to facilitate the effective sharing of health-related information and desired health promotion.

In our study, the findings of SA combined with the relevant analysis of the topic model can enhance our understanding of how public emotions have evolved in response to the launching of COVID-19 re-opening policies. These findings necessitate recalibrated health communication frameworks. The policy reversal’s abruptness, exemplified by zero-COVID discontinuation, amplified public apprehension, underscoring governance imperatives for phased transparency (35). Institutional channels must prioritize real-time dissemination of evidence-based protocols to counter misinformation vectors, particularly during medication scarcity crises (36). Multimodal outreach strategies integrating authoritative media partnerships (37) and algorithmic misinformation detection could mitigate trust erosion. Crucially, message framing should align with cultural priorities—leveraging familial reunion motifs during holiday travel surges—to enhance policy receptivity (8). Existing infrastructure adaptations (e.g., digital health platforms flagged by “link” or “webpage” mentions) provide scalable templates for crisis-responsive communication systems. While these recommendations stem from the re-opening experience in China, their relevance extends further that future health crises will demand not only rapid responses but also proactive, phased communication frameworks that anticipate public concerns and sustain trust over time. Authorities should combine crisis and emergency risk communication principles with health communication, that is, be first, be right, be credible, express empathy, promote actions, and show respect, through regular transparent updates that acknowledge uncertainty (38).

## 6 Conclusion

This study establishes a multidisciplinary framework linking policy abruptness, emotional dynamics, and cultural context. Methodologically, the integration of LDA and domain-specific lexicons advances social media analytics in non-Western contexts. The results obtained captured nuanced emotional fluctuations and linked them to evolving policy-related topics discussed on Weibo, revealing the co-evolution of emotions and issues during a critical period. Substantively, our findings caution against one-size-fits-all communication strategies during pandemics: while rapid deregulation may be epidemiologically justified, its success hinges on preemptive narrative-building to align public expectations. For policymakers, the results suggest several concrete strategies. First, adopting phased and anticipatory communication, rather than abrupt policy shifts, can potentially mitigate fear and disgust. Second, ensuring transparent and frequent updates on risks, medical resources, and policy rationales helps sustain public trust. Moreover, leveraging social media platforms not only as channels for top-down announcements but also for two-way engagement with citizens can enhance responsiveness and reduce misinformation.

However, this study has certain limitations. The method of emotion extraction requires further refinement, as the lexicon-based approach employed has inherent constraints, particularly, some words possess multiple meanings, which may lead to inaccuracies in emotional classification. This problem is also a noted limitation in previous research (17, 39). Additionally, we conducted SA on the overall Weibo data without regional classification. In fact, central cities may exhibit more pronounced emotional fluctuations than peripheral areas, as residents in central cities usually use social media more frequently (17, 40). Therefore, the lack of regional classification may impede our ability to accurately represent the true sentiments of all netizens. Future research endeavors are suggested to overcome the above-mentioned limitations, deepen the integration of SA and LDA into understanding public health-related issues, and explore longitudinal sentiment tracking across policy lifecycles.

## Data Availability

Publicly available datasets were analyzed in this study. This data can be found here: the datasets analyzed in this study consist of publicly available social media posts from Weibo. Due to platform restrictions and privacy considerations, the raw data cannot be openly shared or deposited in a public repository. However, the data can be accessed directly from the Weibo platform (https://m.weibo.cn/) using the specified keywords and time frame described in the Methods section. The data sets generated and analyzed during this study are available from the first author (XN) on reasonable request.
